# Optimization of grape artificial hybrid pollination technology process and the effect of different male parents on the fruit setting rate

**DOI:** 10.3389/fpls.2025.1660290

**Published:** 2025-09-22

**Authors:** Ruijin Zhou, Ruxin Gai, Wanying Liao, Qiujie Wu, Jiahui Cheng, Yongmu Li, Xiucai Fan, Guirong Li

**Affiliations:** ^1^ School of Horticulture and Landscape Architecture, Henan Institute of Science and Technology, Xinxiang, Henan, China; ^2^ Zhengzhou Fruit Research Institute, Chinese Academy of Agricultural Sciences, Zhengzhou, Henan, China; ^3^ Henan Province Engineering Research Centers of Horticultural Plant Resource Utilization and Germplasm Enhancement, Xinxiang, Henan, China

**Keywords:** grape, artificial hybridization, parental selection, pollination technology, pollen viability, fruit setting rate

## Abstract

Grape (*Vitis vinifera* L.) is a globally paramount fruit crop, exerting a pivotal role in China’s agricultural production. However, the grape industry confronts core bottlenecks: centralized table grape listing resulting in recurrent oversupply, underdeveloped independent brand development, scarcity of internationally recognized cultivars, and suboptimal crossbreeding efficiency (e.g., concentrated 6 - 10-day flowering periods, complex flower ear structures, and stringent technical requirements for emasculation/pollination). To tackle these industry and breeding challenges, this study refined artificial hybridization pollination protocols and systematically analyzed pollen viability and hybridization affinity of nine male parents, using ‘Shine Muscat’ (*Vitis labrusca* × *Vitis vinifera*) grape as the female parent. Key findings revealed that pollen collection 1–2 days before messenger flower bloom optimizes viability, Ultra-low -80°C storage sustained pollen viability optimally. Precise early-stage anther removal and three-day pollination post stigma mucus secretion elevate the fruit set rate by 15%–20%. Hybrid combinations exhibited significant variations in fruit set rate (27.8%–62.3%), with ‘Moldova’ grape achieving the highest rate of 62.3%. A standardized process integrating parental selection, pollen management, emasculation-pollination, and data recording was established, elevating hybridization success rates by over 40%. This study delivers a scalable solution for grape cultivation.

## 1 Introduction

Grapes (*Vitis vinifera* L.) are among the most important fruit crops globally and hold profound socioeconomic significance. As a globally recognized core origin center of grape germplasm resources (Li et al., 2014), China regards grapes not only as a key driver of agricultural output growth but also as a critical underpinning for the implementation of the national Rural Revitalization Strategy, and it has further consolidated its position as the world’s largest producer and consumer of table grapes (Atak and Sen, 2021; Yuldashev and Iminov, 2022). In recent years, driven by both advances in cultivation technology and attractive economic returns, grape cultivation has expanded from traditional advantageous regions to all provinces across the country, effectively fueling the continuous expansion of the industry scale. However, the rapid growth in planting area and output has also exposed prominent issues: the concentrated marketing of table grapes leads to periodic oversupply, resulting in volatile market prices; more critically, the development of China’s independent grape brands lags behind, and there is a lack of core cultivars with international recognition—a situation that severely hinders the enhancement of the grape industry’s core competitiveness (Tao et al., 2012).

Crossbreeding is an irreplaceable core technical method for grape cultivar improvement, playing an indispensable role in boosting grapes’ resistance to biotic and abiotic stresses, optimizing fruit quality, and unleashing yield potential (Park et al., 2016; Chen et al., 2024). Since the 1950s, China has achieved substantial breakthroughs in grape crossbreeding: interspecific hybridization between *Vitis amurensis* and Eurasian grape cultivars has yielded cold-resistant wine grape cultivars, including ‘Beichun’ and ‘Beimei’ (Lu and Liu, 2013; Duan et al., 2019); similarly, intraspecific hybridization among Eurasian grape cultivars has produced high-quality table grape cultivars such as ‘Jingzaojing’ and ‘Zaomeigui’ (He et al., 2007; Liu et al., 2023).

Despite these achievements, the efficiency and large-scale application of crossbreeding remain constrained by long-standing technical bottlenecks (Randhawa et al., 1982; Liu et al., 2024). Grapes have a concentrated flowering period (≈10 days) and complex inflorescence structures (each containing over 500 flowers), necessitating extensive artificial emasculation and pollination efforts over a narrow window. Emasculation requires precision removal of stamens from millet-sized flower buds without damaging the stigma—a highly labor-intensive process, as each inflorescence takes several hours to process, and even minor mistakes can result in total hybridization failure. Additionally, pollen viability is highly sensitive to external factors (Wang et al., 2022a), while genotype-dependent differences in cross-compatibility further amplify uncertainty in breeding results (Migicovsky et al., 2016; Xu, 2023; Tan et al., 2024). These unresolved challenges not only limit improvements in breeding efficiency but also hinder the development of cultivars with independent brands and international competitiveness, creating an urgent need for targeted technological innovations to break this impasse.

This study addresses key issues in the industry and breeding field through systematic innovative exploration and technical protocol optimization. The core work includes: taking ‘Shine Muscat’ (*Vitis labrusca* × *Vitis vinifera*) as the female parent—though this cultivar has prominent economic value, research on it in large-scale crossbreeding remains relatively limited; by systematically evaluating the pollen viability of nine male grape cultivars, this study effectively fills the gap in research on parental compatibility of this important female parent line; for the first time, conducting comparative experiments on pollen germination rates focusing on three interacting key factors (pollen collection time, storage temperatures: 4°C vs. ultra-low temperature of -80°C, and storage duration: 0–7 days), providing quantitative basis for precise pollen management—a field that has long lacked systematic data support; simultaneously optimizing core breeding operational links (such as early-stage precise anther removal and timed pollination after stigma mucus secretion), effectively solving the problem of high failure rates in traditional emasculation-pollination processes; furthermore, by analyzing the fruit set rates and seed traits of all hybrid combinations, establishing a standardized and scalable artificial cross-pollination protocol. These key technological innovations not only effectively break the bottleneck of low breeding efficiency caused by concentrated flowering periods but also lay a solid technical foundation for the breeding of new grape cultivars.

## 2 Materials and methods

### 2.1 Materials

This study was conducted from April 2024 to May 2025 at the Grape Germplasm Resources Garden at Henan Institute of Science and Technology in Xinxiang City, Henan Province. The test female parent was ‘Shine Muscat’ grape (*Vitis labrusca* × *Vitis vinifera* ‘Shine Muscat’). The paternal cultivars comprised nine locally characteristic grapes (Liu et al., 2016): ‘Gold Finger’, ‘Moldova’, ‘Shenzhou Red’, ‘Queen Nina’, ‘Giant Rose’, ‘Hotan Red’, ‘Romantic Beauty’, ‘Sweet Sapphire’, and ‘Zuijinxiang’ grape. The characteristics of these nine cultivars were shown in **Table 1**.

**Table 1:** Characteristics of grape cultivars used for testing.

Parent	Cultivar	Berry color	Berry size	Resistance
Female parent	‘Shine Muscat’ grape	Green	Medium to large (8–12 g)	Poor stress resistance, disease resistance, and insect resistance
Father 1	‘Gold Finger’ grape	Green	Medium (8–10 g)	Cold resistant, disease resistant, drought resistant, and flood resistant
Father 2	‘Moldova’ grape	Purplish black	Medium to small (5–8 g)	Cold resistant, disease resistant, and drought resistant
Father 3	‘Shenzhou Red’ grape	Red	Medium to large (8–12 g)	Disease-resistant
Father 4	‘Queen Nina’ grape	Red	Extra Large (13–17 g)	Disease-resistant
Father 5	‘Giant Rose’ grape	Purplish black	Large (9.5–12 g)	Disease resistance and high temperature tolerance
Father 6	‘Hotan Red’ grape	Red	Medium (6–10 g)	Cold resistant, drought resistant, salt alkali resistant
Father 7	‘Romantic Beauty’ grape	Red	Large (10–13 g)	Disease-resistant
Father 8	‘Sweet Sapphire’ grape	Purplish black	Large (10–14 g)	Cold resistant and drought resistant
Father 9	‘Zuijinxiang’ grape	Green	Large (10–15 g)	Disease-resistant

### 2.2 Methods

#### 2.2.1 Determination of pollen viability in paternal grape cultivars

Pollen is crucial in grape hybrid breeding, and pollen vitality significantly impacts breeding outcomes. Pollen quality is an essential indicator in grape production. In this study, 9 different grape cultivars were selected as hybrid male parents. Different pollen collection times (continuous picking 7 days before pollen opening), storage times (0, 5, and 7 days), and storage temperatures (4°C and -80°C) were used to grind the pollen of different male parent grapes. The pollen was passed through a sieve and evenly spread on a germination bed (200 g·L^-1^ sucrose + 7 g·L^-1^ agar + 0.08 g·L^-1^ boric acid medium (Kelen et al., 2003) or *in vitro* culture. A disposable plastic culture dish (6 cm × 6 cm) was used to make the germination bed. Pollen germination was observed at different culture times (1, 2, 3, 5, and 6 h). The pollen germination force was observed under an optical 10 × 40 upright biological microscope (UB103i; Chongqing Aopu Optoelectronic Technology Co., Ltd.). The differences in pollen vitality among different male parents were analyzed, and the effects of different pollen collection times and storage conditions on pollen vitality were investigated.

#### 2.2.2 Hybrid pollination technique

The preparation for hybrid pollination involved collecting and storing the male parent pollen, determining pollen vitality, castrating the female parent flower, performing pollination, and managing the plant after pollination. The fruit setting rate was calculated as follows: fruit setting rate% = number of fruits set/number of hybrid pollination × 100%.

#### 2.2.3 Data processing and analysis

**Figure 1:**
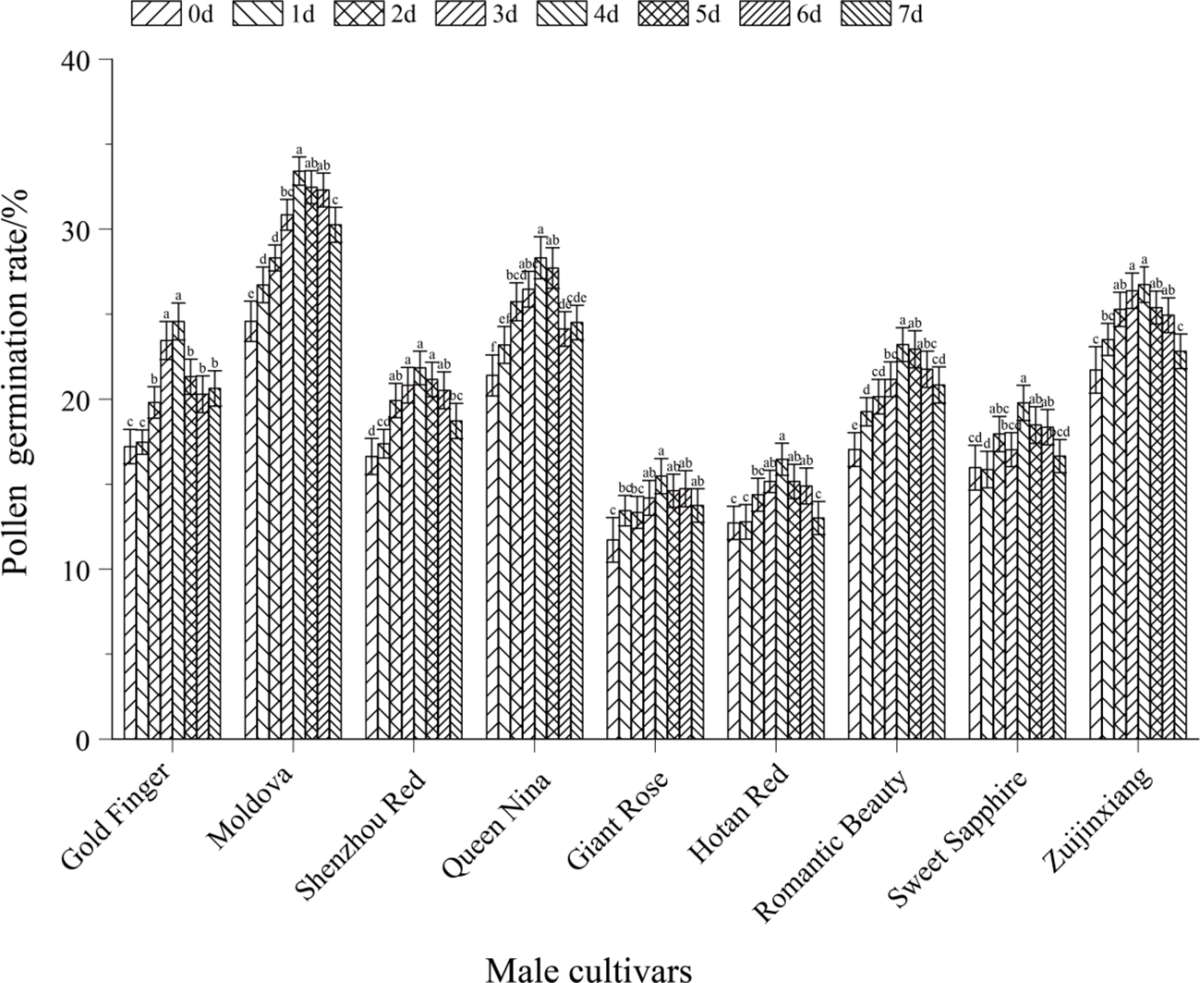
Effect of different pollen harvesting times on pollen viability in paternal parent grape. The figure presented the pollen germination rates of various paternal grape cultivars (Gold Finger, Moldova, Shenzhou Red, Queen Nina, Giant Rose, Hotan Red, Romantic Beauty, Sweet Sapphire, Zujinxiang) across different pollen harvesting time points (0d, 1d, 2d, 3d, 4d, 5d, 6d, 7d). Different lowercase letters above the bars denote significant differences in pollen germination rate among different harvesting times for the same cultivar (*P* < 0.05).

**Figure 2:**
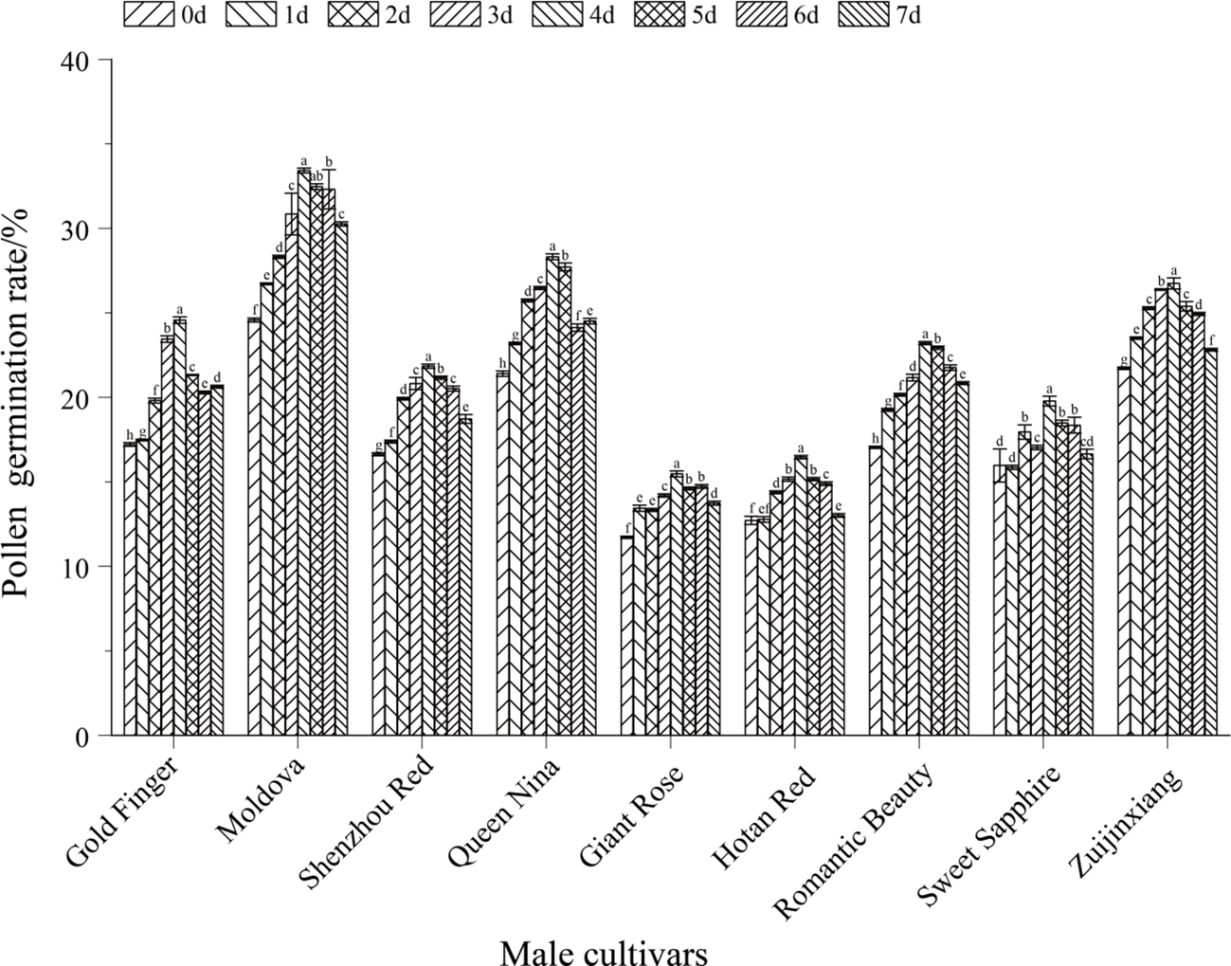
Effects of different storage temperatures, storage Times, and incubation times on pollen viability in paternal parent grape. different colored bars correspond to the pollen germination rates at 0 day (0d), 1 day (1d), 2 days (2d), 3 days (3d), 4 days (4d), 5 days (5d), 6 days (6d), and 7 days (7d) of storage, respectively; different lowercase letters above the bars indicate that there are significant differences in pollen germination rates among different storage days for the same grape cultivar (*P* < 0.05).

Data were organized using Microsoft Excel 2010, and results were expressed as mean ± standard deviation (mean ± SD). Statistical analyses and graphing were conducted using GraphPad Prism 10.1.2 software. One-way analysis of variance (ANOVA) followed by Duncan’s multiple comparison test was applied to assess significant differences among experimental groups, with the significance level strictly set at *P* < 0.05. Consistent with this statistical criterion, different lowercase letters labeled above the bars in the figures (e.g., **Figures 1**, **2**) indicated significant differences in the measured indices (e.g., pollen germination rate, fruit setting rate) among different treatment groups or cultivars (*P* < 0.05).

## 3 Results

### 3.1 Optimizing of preparations for cross-pollination

#### 3.1.1 Observations on flowering characteristics of grapes

After maturation of the pollen and development of the pistil, grapes were cross-pollinated mainly by closed-flower fertilization, that is, pollination in the cap before the corolla opened (Ban et al., 2016). The early and long flowering periods of plants are influenced by local climatic conditions and cultivars. The flowering period of grape in Henan Province is mainly concentrated at the end of April to the first half of May, and the flowering period lasts 7–14 days. First, the top or the middle of the spikelet flower opens, and then the opening proceeds gradually to the middle and the top of the inflorescence. At the time of flowering, the five lobes at the base of the corolla are revolute and fall off, exposing the androgynophore. The anthers split to disperse yellow pollen, which is spread by wind and insects. It opens at 7–9 a.m. every day, and the whole inflorescence finishes opening in about 3–4 days.

#### 3.1.2 Specifications for inflorescence thinning of parent plants

Inflorescence thinning of the parent material is generally carried out 10–15 days before flowering, particularly when more and larger inflorescences were present, to concentrate the nutrient supply, improve the rate of fruit set and fruit quality, and ensure the quality of pollination. When preparing to remove male flowers, weak and abnormally developed inflorescences are thinned out first. It is recommended to leave only one stout inflorescence per fruiting branch within a branch group.

#### 3.1.3 Preparation of hybridization tools

The following tools were used for pollen collection: a sulfate paper for dispersing pollen, a mortar for grinding pollen, a sieve for sifting pollen, and a centrifuge tube for collecting pollen. The tools used for cross-pollination included 25 cm × 16 cm hybrid bags made of folded waste newspaper, paper pins, spray cans, pins for sealing paper bags after desexing or pollination, hanging swabs (6 cm × 4 cm), marking pens, brushes for pollination, sterilizing alcohol, and so forth.

#### 3.2 Optimization of pollen collection and storage techniques for parent plants

**Figure 3:**
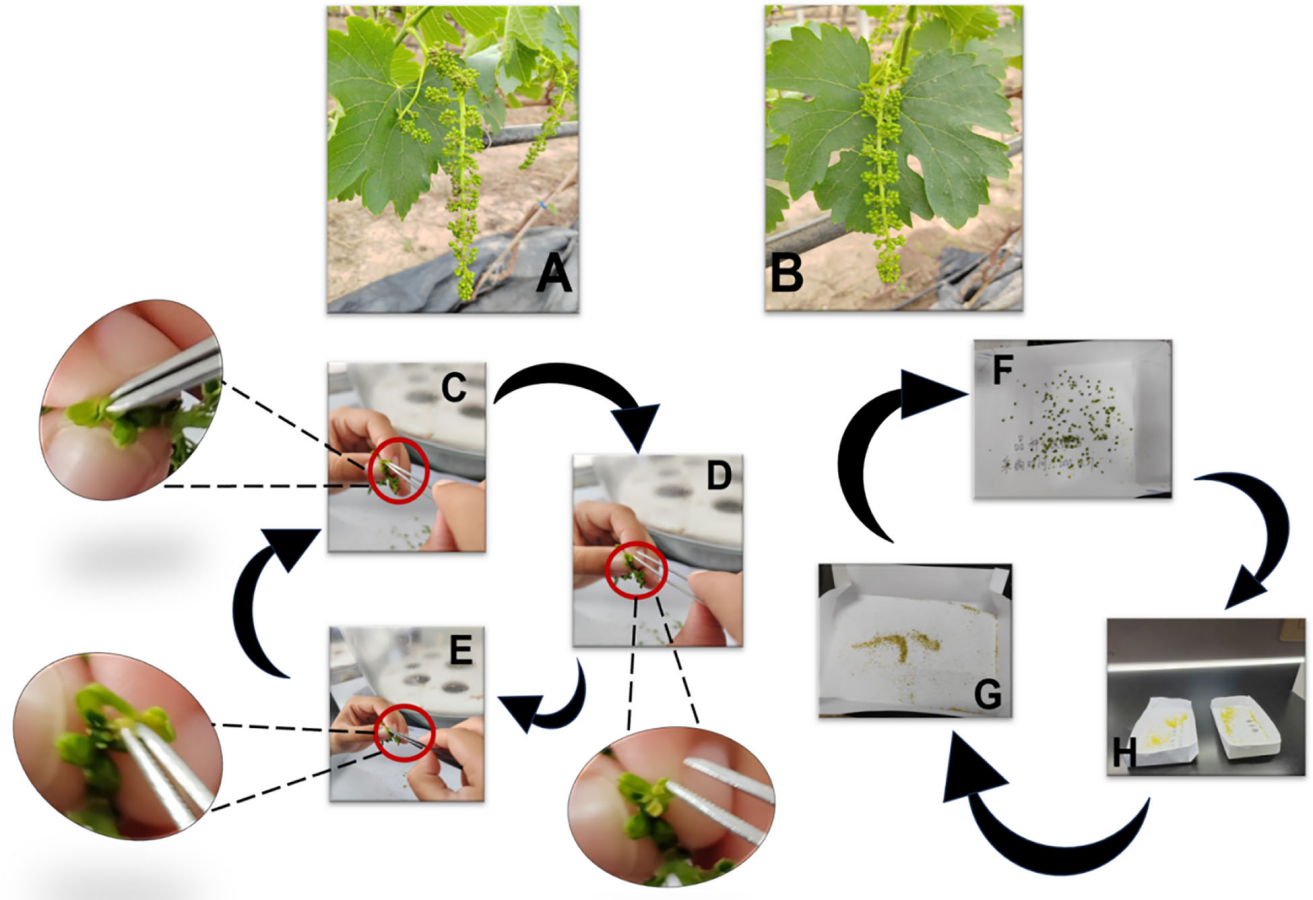
Process of grape pollen collection. **(A, B)** Morphological signals for determining the optimal pollen collection period (e.g., yellowing of flower caps and initial blooming of messenger flowers, which indicate mature pollen ready for collection). **(C–E)** Anther stripping process: sequentially peeling off green flower caps **(C)**, carefully removing anthers from flower buds **(D)**, and manually eliminating impurities (e.g., residual flower cap fragments) to ensure pollen purity **(E)**. **(F–H)** Pollen release (loose powder) process: placing anthers in a ventilated environment at ~28°C **(F)**, air-drying anthers for 24 h to reduce moisture **(G)**, and facilitating the release of mature pollen grains from dried anthers **(H)**.

The pollen collection of parent grapes is strictly decided by their growth status. As shown in **Figures 3A, B** (morphological signs for optimal timing), pick pollen when the flower spike stretches, flower cap turns yellow, and messenger flower just blooms. These traits confirm the pollen’s physiological maturity which is a key factor for later viability. During collection, select flower spikes with full grains and larger florets first. Cut the chosen spike with sterile scissors, remove its top (to discard underdeveloped tissues), and put the rest into a clean self-sealing bag to finish picking. After picking, move spikes to Ziplock bags and store in a foam box with ice packs. This keeps spikes moist, prevents pollen cell damage from water loss, and maintains pollen quality for lab work. Send the box to the lab right after field collection to avoid harm from long exposure to ambient conditions (Li, 2013). In the lab, take spikes out carefully and place in clean, dry white cardboard boxes. Mark each box’s inner wall with cultivar name and picking time (waterproof marker) to avoid sample confusion. Strip anthers and remove impurities per **Figures 3C–E**: First, take one spike from the Ziplock bag at a time, closing the box lid immediately; Second, use pointed sterile tweezers to remove flower clusters, then peel off green caps from each floret (**Figure 3C**, no anther damage); Third, separate anthers from buds (**Figure 3D**), collect into labeled boxes, and hand-pick residual caps/impurities (**Figure 3E**) to avoid contamination. After one cultivar’s anther stripping, replace tweezers or sterilize them (soak in 75% alcohol for 5–10 minutes, wipe with sterile alcohol cotton, air-dry in a laminar flow hood) to prevent cross-contamination. To release pollen, place anther-containing boxes under a sterile lamp in a well-ventilated area (28 °C). Dry continuously for 24 hours (**Figures 3F–H**): first, let anthers lose moisture gradually (**Figure 3F**); after 24 hours, brittle anther walls crack, releasing pollen (**Figures 3G, H**). This drying step ensures the pollen is fully separated from the anther tissues, forming pure pollen samples ready for later sieving and storage.

**Figure 4:**
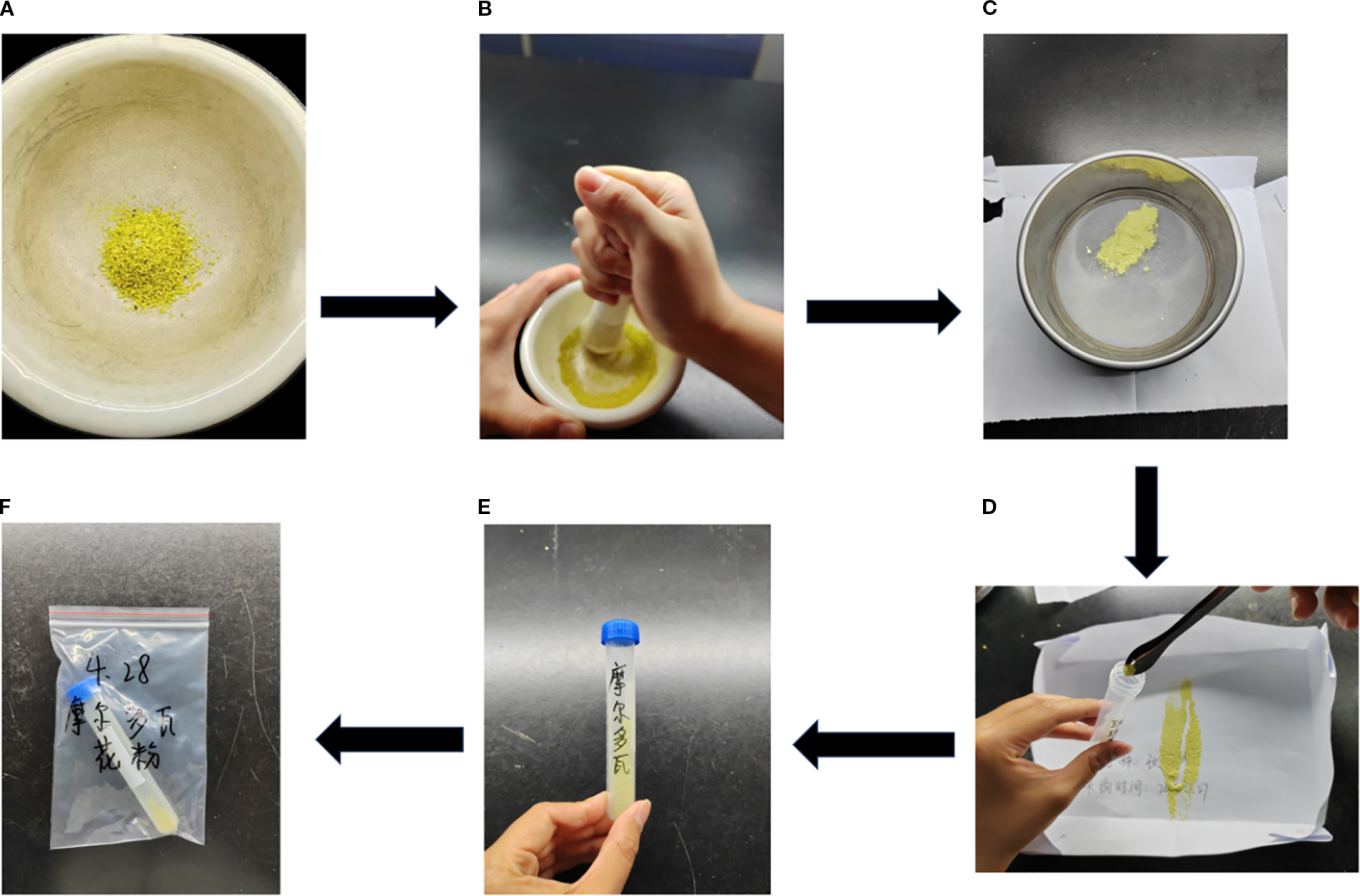
Process of grape pollen dispersal, collection, and storage. **(A)** Loose powder poured into a mortar and pestle. **(B)** Pollen grinding.**(C)** Pollen sieving. **(D)** Pollen dispensing with a medicine spoon. **(E)** Labeling freezer tubes (cv. ‘Moldovan’ grape). **(F)** Pollen stored in self-sealing bags in a refrigerator (cv. ‘Moldovan’ grape).

Pollen storage is crucial for successful grape cross-pollination. Good storage ensures that pollen viability is not lost quickly, and pollination is ensured. The parent pollen storage operation included the following steps. When the anthers were completely dry, they were ground to a fine powder with force and then sifted with a sieve. The edges of the sieve mesh were tapped with fingers to ensure that all the pollen were sifted into the cardboard box. Both the mortar and the sieve were cleaned and sterilized after use, disinfected with 75% alcohol, and put in the oven for drying. Finally, all the pollen from the carton were put into 5-mL freezer tubes (the bottom of the carton flicked with the finger to ensure that the pollen is not wasted) using a spoon. The tubes were marked with the name of the species and the time of year, and stored in the refrigerator for later use (**Figure 4**).

### 3.3 Key factors influencing pollen viability of paternal parents

#### 3.3.1 Effect of pollen collection time on pollen viability of paternal grape cultivars

**Figure 5:**
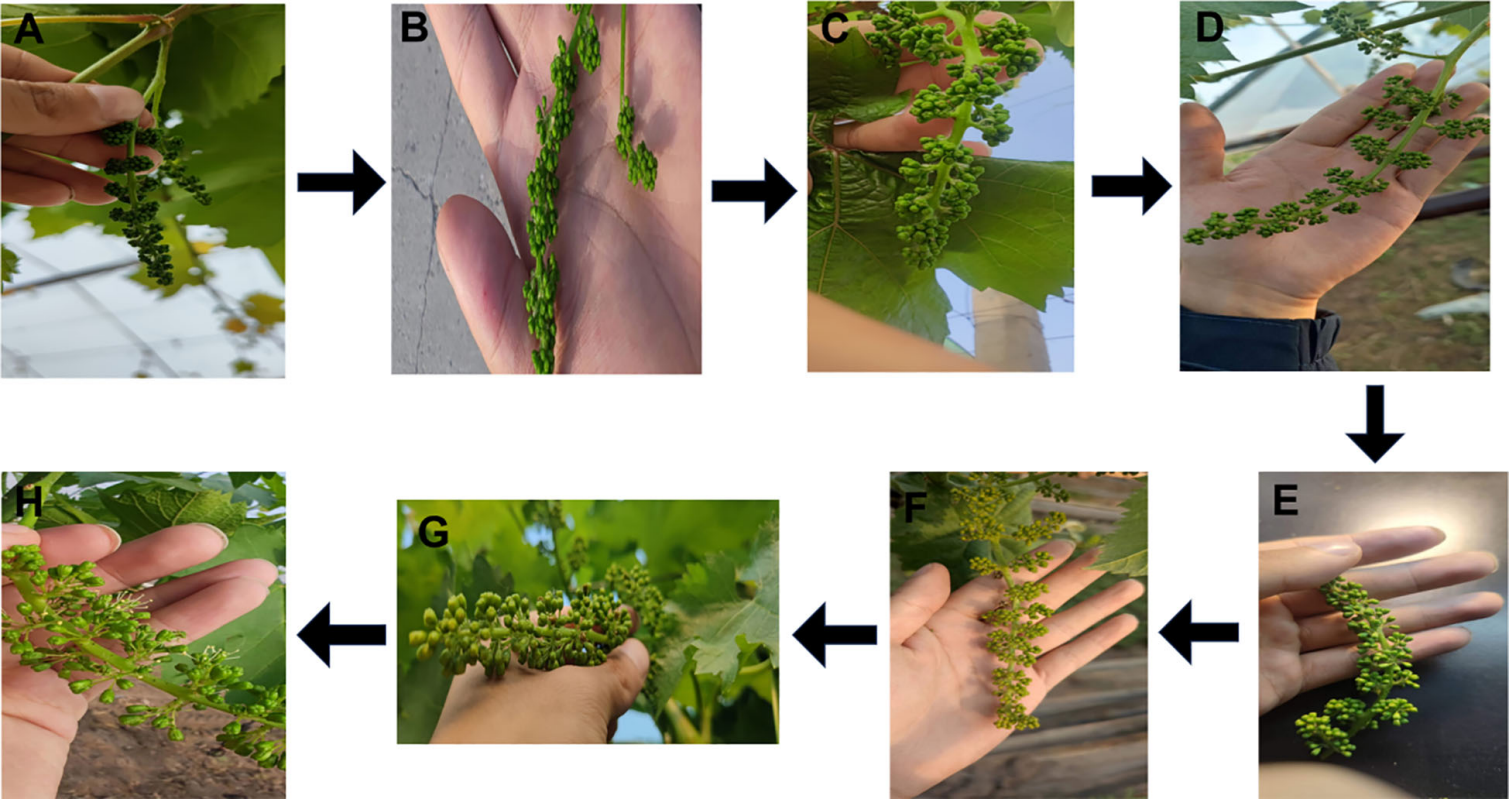
Effect of different pollen harvesting times on pollen viability in paternal parent grape and growth status of flower spikes. **(A)** Growth status of flower spikes at 7 days before pollen picking. **(B)** Growth status of flower spikes at 6 days before pollen picking. **(C)** Growth status of flower spikes at 5 days before pollen picking. **(D)** Growth status of flower spikes at 4 days before pollen picking. **(E)** Growth status of flower spikes at 3 days before pollen picking. **(F)** Growth status of flower spikes at 2 days before pollen picking. **(G)** Growth status of flower spikes at 1 day before pollen picking. **(H)** Growth status of flower spikes at 0 day (on the day of) pollen picking.

The pollen viability of paternal grape cultivars exhibited a unimodal variation trend (first increasing and then decreasing) with pollen collection time (from 7 days before flowering to the day of flowering). The pollen viability peaks of all tested paternal cultivars were concentrated at 1–2 days before flowering (the initial blooming stage of messenger flowers). At this stage, pollen viability was significantly higher than that at other collection times (*P* < 0.05). This pattern can be fully verified by both morphological observations of flower spikes (**Figure 5**) and quantitative analysis of pollen germination rates (**Figure 1**).

##### 3.3.1.1 Biological morphological characteristics of flower spikes

From the perspective of the biological morphological characteristics of flower spikes (**Figure 5**), paternal flower spikes at different pollen collection times showed distinct developmental differences, providing intuitive evidence for determining the optimal pollen collection time: From 7 days before flowering (**Figure 5A**) to 3 days before flowering (**Figure 5E**): The flower spikes were generally small and thin, with calyptras mainly green and no obvious signs of maturity. At 2 days before flowering (**Figure 5F**): The flower spikes gradually elongated, and some calyptras began to turn yellow, while messenger flowers remained closed. At 1 day before flowering (**Figure 5G**): The flower spikes were plump, calyptras turned significantly yellow, and messenger flowers initiated blooming. The developmental state of floral organs at this stage indicated that pollen had entered the physiological maturity stage. On the day of flowering (**Figure 5H**): Most flowers on the spikes opened, calyptras fell off, and some pollen had been naturally dispersed, which easily led to losses in both pollen quantity and viability.

##### 3.3.1.2 Quantitative analysis of pollen germination rates

Quantitative results of pollen germination rates (**Figure 1**) showed that there were significant differences in pollen viability among the 9 paternal grape cultivars at different collection times (*P* < 0.05). All cultivars exhibited the common pattern of peaking at 1–2 days before flowering, while also showing cultivar-specific characteristics: Taking ‘Moldova’ grape as an example, its pollen germination rate was the highest (33.42%) when collected 1 day before flowering, which was significantly higher than that collected 7 days before flowering (24.58%) and on the day of flowering (28.76%) (P < 0.05). ‘Queen Nina’ and ‘Gold Finger’ grapes showed a similar trend: their pollen germination rates reached respective peaks at 1–2 days before flowering, which were significantly higher than those at 3 days or later before flowering and 6 days or earlier before flowering (P < 0.05), demonstrating strong temporal dependence of pollen viability. The pollen viability of ‘Giant Rose’ and ‘Hotan Red’ grapes was generally low. Their viability peaks also occurred at 1–2 days before flowering, but the peak germination rates were both below 20%. Moreover, at non-optimal collection times, their pollen viability decreased more significantly (*P* < 0.05). For instance, the pollen germination rate of ‘Giant Rose’ was only approximately 10% when collected 7 days before flowering, which was significantly lower than the 24.58% of ‘Moldova’ at the same time point (*P* < 0.05). Additionally, the germination rate of ‘Giant Rose’ on the day of flowering decreased by more than 40% compared to its own peak (*P* < 0.05).

In summary, combining the morphological characteristics of flower spikes (**Figure 5**) and pollen germination rate data (**Figure 1**), it can be clearly concluded that the initial blooming stage of messenger flowers (1–2 days before flowering) is the optimal time for collecting paternal grape pollen. At this stage, pollen grains have completed meiosis, with high physiological maturity, sufficient nutrient accumulation, and stable cell wall structure—thus achieving the highest germination rate. If pollen is collected too early, the pollen will be immature and less viable; if collected too late, pollen viability will decrease significantly due to dispersion and senescence (*P* < 0.05). This result provides dual morphological and physiological evidence for the precise collection of paternal pollen in grape artificial hybrid pollination.

#### 3.3.2 Effects of storage temperature, storage duration, and incubation time on pollen viability of paternal parents

Storage duration has a considerable impact on pollen germination. Under the -80°C condition, the germination rate of most cultivars decreased slightly within 5 days of storage. For example, the germination rate of ‘Moldova’ peaked at 33.42% after 1 day of storage, remained at 23.44% after 5 days (showing no significant difference compared with the 0-day and 1-day storage groups), and even maintained a germination rate of 30.26% after 7 days of storage, which was significantly higher than that of other cultivars in the same period (**Figure 2**).

**Figure 6:**
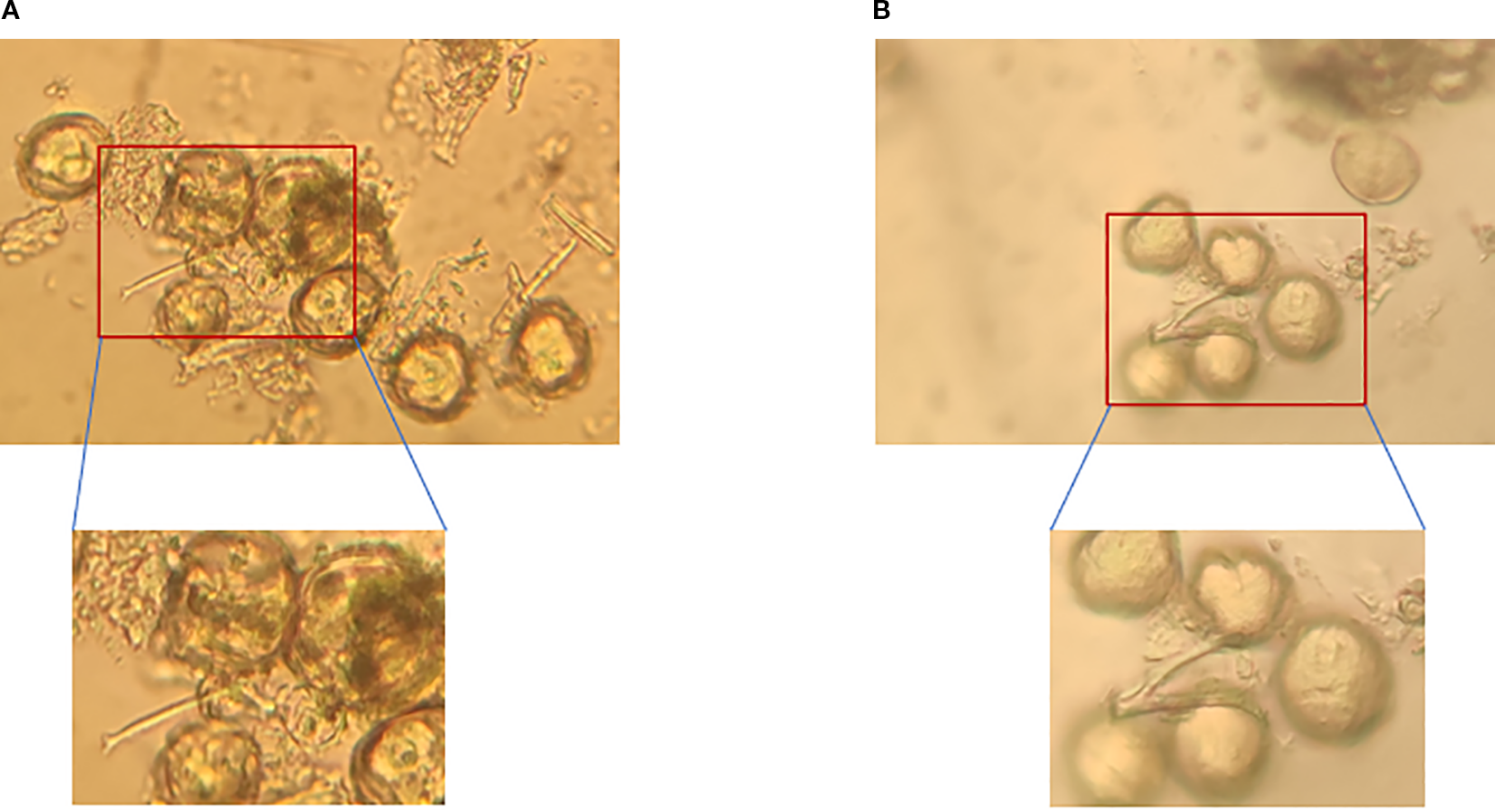
Comparison of pollen germination viability between ‘Queen Nina’ and ‘Moldova’ grapes after low-temperature storage. **(A)** Germination of ‘Moldova’ grape pollen after 7 days of storage at 4°C followed by 3 hours of incubation. **(B)** Germination of ‘Queen Nina’ grape pollen after 7 days of storage at 4°C followed by 3 hours of incubation.

Culture time exerts a dynamic regulatory effect on the recovery of pollen viability, and there were significant differences in germination rates among different culture durations (*P*<0.05). Under short-term culture (1–2 hours), pollen tube elongation was insufficient and metabolism was not activated, resulting in a low germination rate. For instance, after ‘Moldova’ was stored at 4°C for 7 days, its germination rate was only 8.93% under 1-hour culture, which was significantly different from that of the 3–5 hour culture groups (**Figure 6A**, *P*<0.05). When the culture time was extended to 3–5 hours, the metabolic activity of pollen increased significantly, the germination rate reached the peak, and the pollen tubes were the longest. Taking ‘Queen Nina’ as an example: after being stored at -80°C for 7 days, its germination rate under 5-hour culture (18.41%) was nearly 80% higher than that under 1-hour culture (10.36%) (**Figure 6B**, *P*<0.05), which further confirms the rationality of this culture duration.

**Figure 7:**
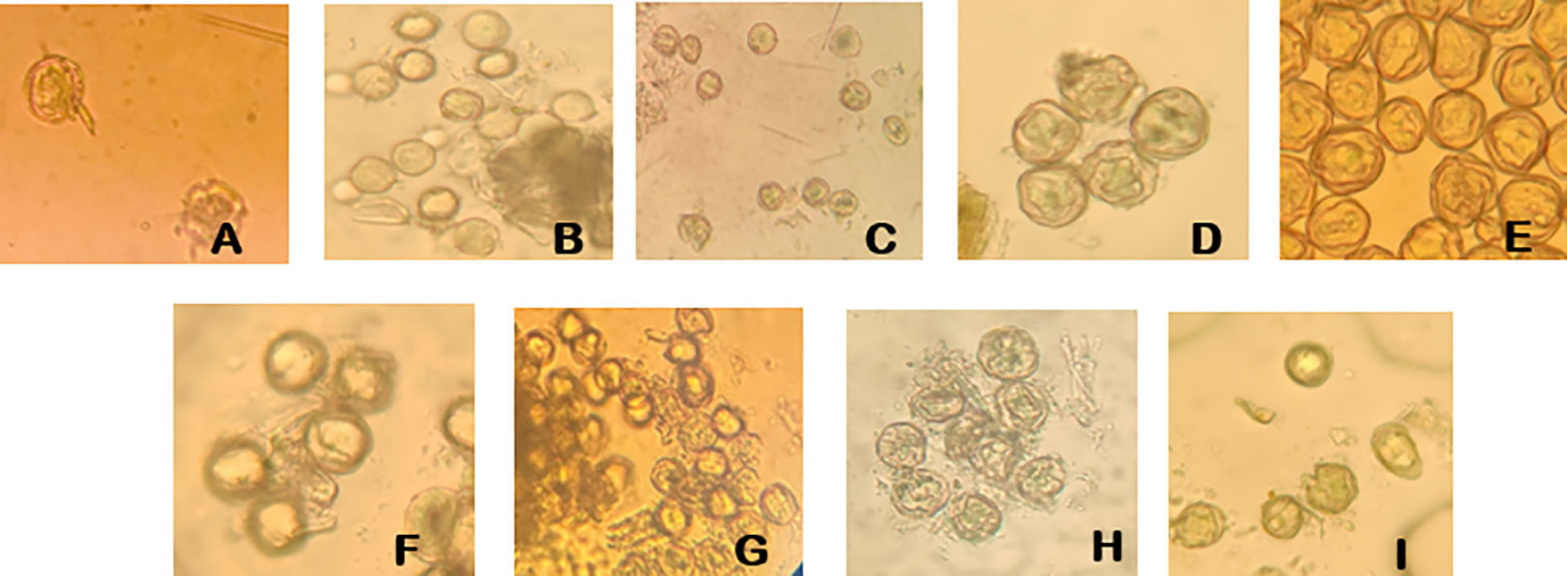
Pollen germination viability of different grape cultivars. **(A)** Pollen germination of ‘Gold Finger’ grape. **(B)** Pollen germination of ‘Moldova’ grape. **(C)** Pollen germination of ‘Shenzhou Red’ grape. **(D)** Pollen germination of ‘Queen Nina’ grape. **(E)** Pollen germination of ‘Giant Rose’ grape. **(F)** Pollen germination of ‘Hotan Red’ grape. **(G)** Pollen germination of ‘Romantic Beauty’ grape. **(H)** Pollen germination of ‘Sweet Sapphire’ grape. **(I)** Pollen germination of ‘Zuijinxiang’ grape.

There were significant genetic differences in the tolerance of different male parent cultivars to storage conditions (*P*<0.05) (**Figure 7**). The variation trends of different cultivars were significantly different, and the germination rates of different cultivars in the same period also showed significant differences (*P*<0.05). Among high-tolerance cultivars, ‘Moldova’ performed the best—it maintained a germination rate of 30.26% even after 7 days of storage at -80°C (the height of the bar for the 7th day was significantly higher than that of other cultivars, *P*<0.05) and could also maintain relatively stable viability under short-term storage at 4°C (within 3 days, the difference from the 0-day group was close to 0.05), making it the preferred male parent for large-scale hybridization. ‘Zuijinxiang’ and ‘Queen Nina’ ranked second: their germination rates remained above 20% within 5 days of storage at -80°C with no significant differences, and ‘Queen Nina’ showed excellent viability recovery under 5-hour culture (exhibiting a significant difference from low-viability cultivars in terms of recovery effect in the same period, *P*<0.05), so it can be used as an alternative highly compatible male parent. Low-tolerance cultivars such as ‘Giant Rose’ and ‘Hotan Red’ had low basic pollen viability and were sensitive to storage (*P*<0.05): for ‘Giant Rose’, when stored at 4°C, its germination rate decreased from 18.2% (0 day) to 10.5% after 3 days (*P*<0.05) and dropped to nearly 0 after 7 days (showing significant differences from the 0-day and 3-day groups, *P*<0.05); for ‘Hotan Red’, its germination rate was less than 15% after 7 days of storage at -80°C (showing a significant difference from ‘Moldova’s 30.26% in the same period, *P*<0.05), so such cultivars should be used with caution.

### 3.4 Refined management of emasculation operations for female parents

**Figure 8:**
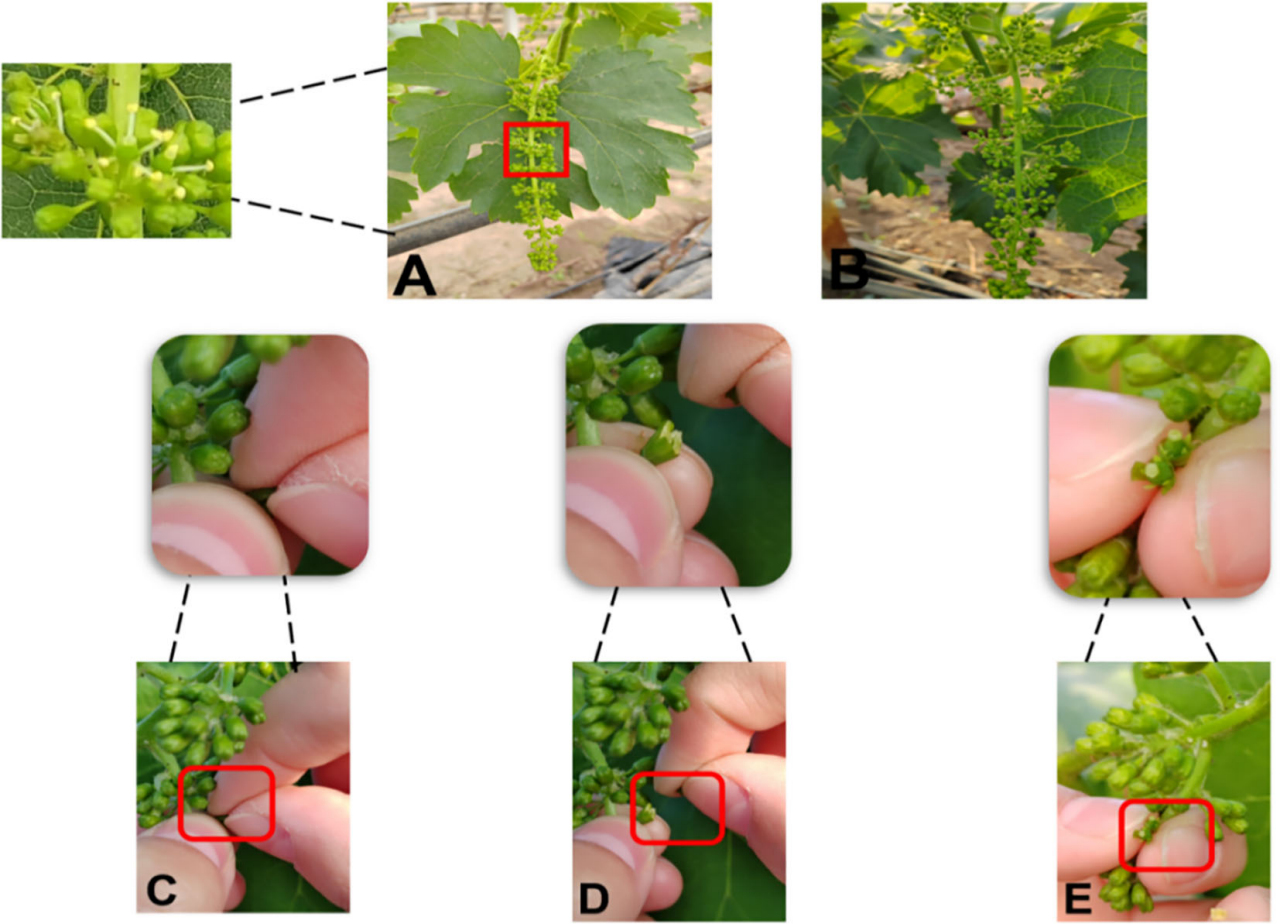
Grape female parent emasculation process. **(A)** Emasculation signal. **(B)** Emasculated stigma. **(C–E)** Emasculation process.

**Figure 9:**
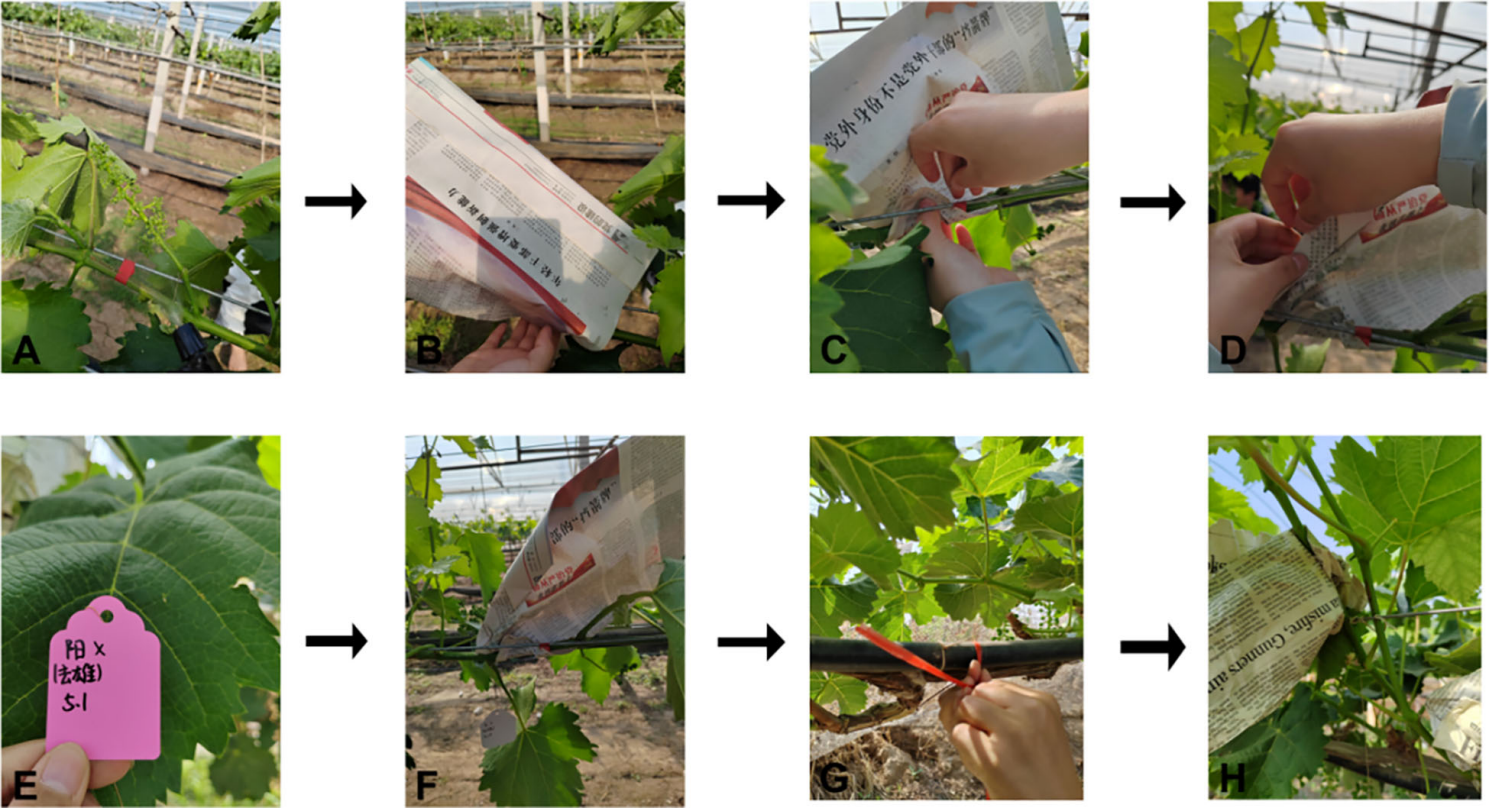
Post-emasculation bagging, fixation, and labeling process for grape female parent. **(A)** Spraying water. **(B)** Bagging. **(C)** Fixing with a pin. **(D)** Fixing with a paper clip **(E)** Marking (maternal, date). **(F–H)** Completing bagging.

Emasculation was conducted cleanly and thoroughly, ensuring anthers were removed without damaging the stigma. The specific operation for female parent emasculation is as follows (**Figures 8**, **9**). First, select flower spikes with messenger flowers blooming (i.e., 1–2 open flowers on the spike, corresponding to **Figure 8A**’s emasculation signal) for the procedure. Before emasculation, all already opened flowers must be removed to prevent self-pollination—this removal should be done from the base of the flower spike. Gently shake the spike by hand to eliminate underdeveloped florets; if the spike has side branches, prune them off, leaving only one main branch. Next, cut off the front one-third of the spike, then start the emasculation operation.

When removing anthers, begin from the base of the spike: use one hand to stabilize the spike, and with the thumb and index finger (or tweezers) of the other hand, peel off the flower caps (**Figures 8C–E**, the detailed emasculation process). Apply gentle force to avoid harming the ovary, and take care not to touch the white upper part of the ovary to prevent disrupting its growth and development. After processing the entire spike, promptly clear any residual anthers to avoid self-pollination, ensuring the stigma remains intact (**Figure 8B**, the emasculated stigma).

Immediately after anther removal, spray water with a spray bottle to maintain moisture (**Figure 9A**), and quickly bag the spike to prevent stigma contamination (**Figure 9B**). When bagging, prop open the newspaper bag to provide sufficient space for spike growth and avoid rupture from contact with damp paper. Wrap one side of the bag around the vine and secure it with a large headpin (**Figure 9C**); fold the other side tightly to block foreign pollen and fix it with a paper clip (**Figure 9D**)—foreign pollen intrusion would lead to significant experimental errors. Finally, hang a tag on the fruiting branch, marking the hybrid combination and emasculation date (**Figure 9E**). Adjusting the hybridization bag to ensure no mechanical squeeze on the flower spike, inspecting the bag seal for potential gaps that may allow foreign pollen intrusion, and presenting the final stable bagging state where the flower spike is properly accommodated without contact with the bag interior (**Figures 9F–H**).

### 3.5 Optimizing of cross-pollination techniques

**Figure 10:**
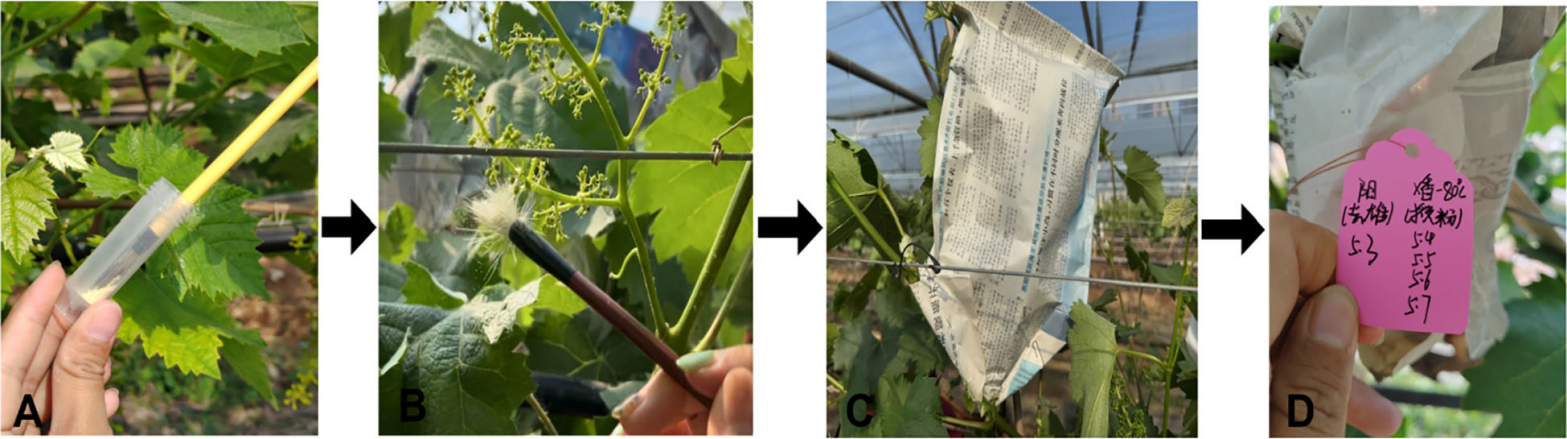
Process of artificial hybrid pollination for grape. **(A)** Dipping pollen with a fine brush. **(B)** Conducting pollination. **(C)** Bagging after pollination. **(D)** Labeling (maternal and paternal dates).

The whole process of grape cross-pollination is shown in **Figure 10**.

#### 3.5.1 Pollination timing and operational practices

Pollination was carried out during the best pollination period. Before pollination, the wind direction was carefully observed so as not to let the pollen be blown away by the wind. Blackened or necrotic stigmas due to improper operation and other reasons during the previous de-masculinization were removed. When pollen was taken out of the bag, the bag was propped up on both sides with hands to avoid mechanical damage to the flower spikes, which could lead to the shedding of the fruits. A fine brush was dipped into the pollen of the parent (because pollen material was precious and care was taken to avoid wastage). A piece of newspaper was placed underneath to catch any falling pollen. The brush or pollination stick was held in one hand, and the pollen was scattered by shaking the fine brush or the pollination stick (or by dipping the pollen directly into the stigma). When pollinating the stigma of the female flower spikes, any residual pollen on the newspaper was used by lightly flicking the bottom of the newspaper to allow the pollen to disperse the stigma. Pay attention not to touch the flower spikes to prevent fruit abscission. After pollination, immediately cover with a new hybridization bag to prevent other pollen from contaminating the female parent. When bagging, use hands to open the newspaper bag to ensure enough space for fruit growth in the future. Finally, on the tag hung during emasculation, mark the male parent and pollination time completely. During pollination, prepare more pollination sticks and fine brushes. Pollination sticks or fine brushes of different cultivars should not be used crosswise to avoid pollen contamination and affect the authenticity of hybridization. Note that when changing pollination cultivars, disinfect hands and pollination tools to prevent cross-contamination of pollen. The pollination process was conducted by two people working together to ensure that each seed was coated with pollen and the quality of the final seed harvest was maintained (Yan et al., 2014; Liang and Liang, 2016; Fu and Geng, 2014).

#### 3.5.2 Marking systems and records management

Flowering branches that were bagged for pollination were tagged and labeled for future seed testing. As the tags were small, detailed information was avoided; hybrid combinations and other related contents were abbreviated using symbols or the first letters of the parent cultivars. In addition to tagging, a detailed hybridization file was maintained. This file included all kinds of situations and data of hybrid combinations in detail (hybrid combination number, parent’s name, the time of pollen collection or storage used, the date and number of times of pollination, the development of hybrid seeds, the number of effective seed collection, etc.) to facilitate future research.

#### 3.5.3 Pollination time and frequency

**Figure 11:**
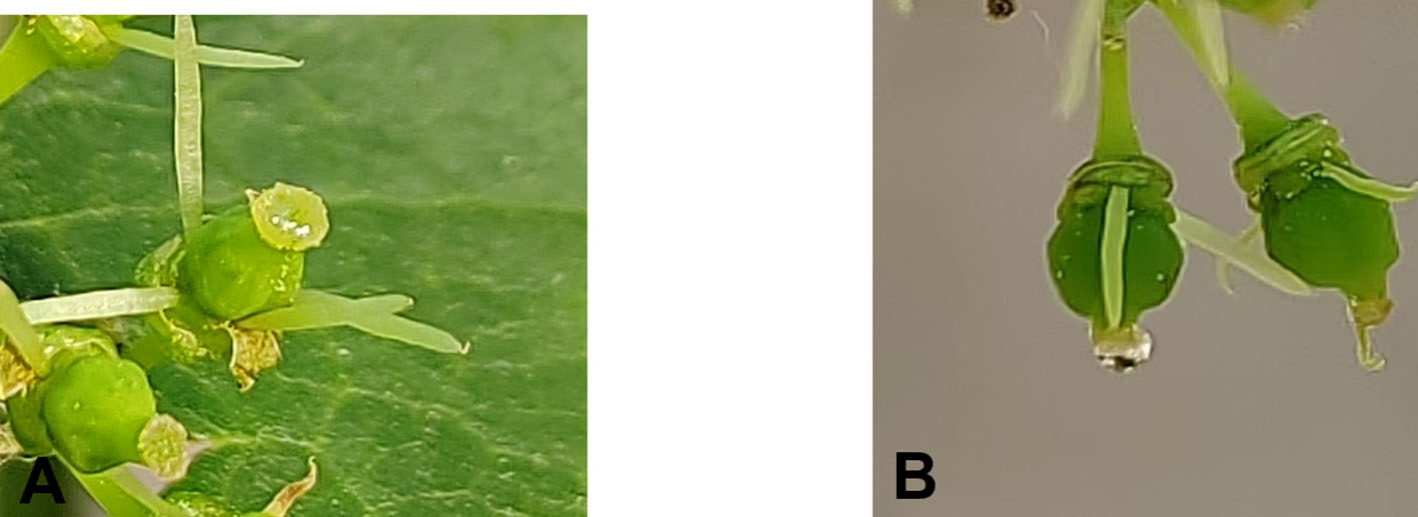
Developmental stages of stigma mucus in grape female parent. **(A)** Early stage of stigma mucus. **(B)** Mature stage of stigma mucus.

The female parent was determined for the best pollination period (after the stigma fully matured to secrete water droplet-like mucus, as shown in (**Figure 11**). The sunny and windless morning 07:00 - 10:00 was the best time to carry out the pollination process. Pollination was carried out continuously for 3 days and repeated three times to ensure successful pollination and increase the pollination rate. A higher number of pollinations can improve fruit set, and tagging hybrid fruit branches helps track the combination and date of pollination. After three pollinations to ensure accuracy, one more pollination can be prepared.

#### 3.5.4 Post-pollination management

The tips of new shoots were wiped off after pollination to balance nutrient growth and reproductive growth, reduce nutrient loss, and promote the enlargement of flower spikes and neat flowering. After pollination, the bag used to protect the pollinated flower was checked for damage and replaced in time. The management of the parent stock plant was improved according to the cultivation management practices of the grape. The hybrid bags were removed after 7 days to ensure light exposure of hybrid fruits while checking for fruit set.

### 3.6 Effect of paternal parents on hybridization outcomes

**Figure 12:**
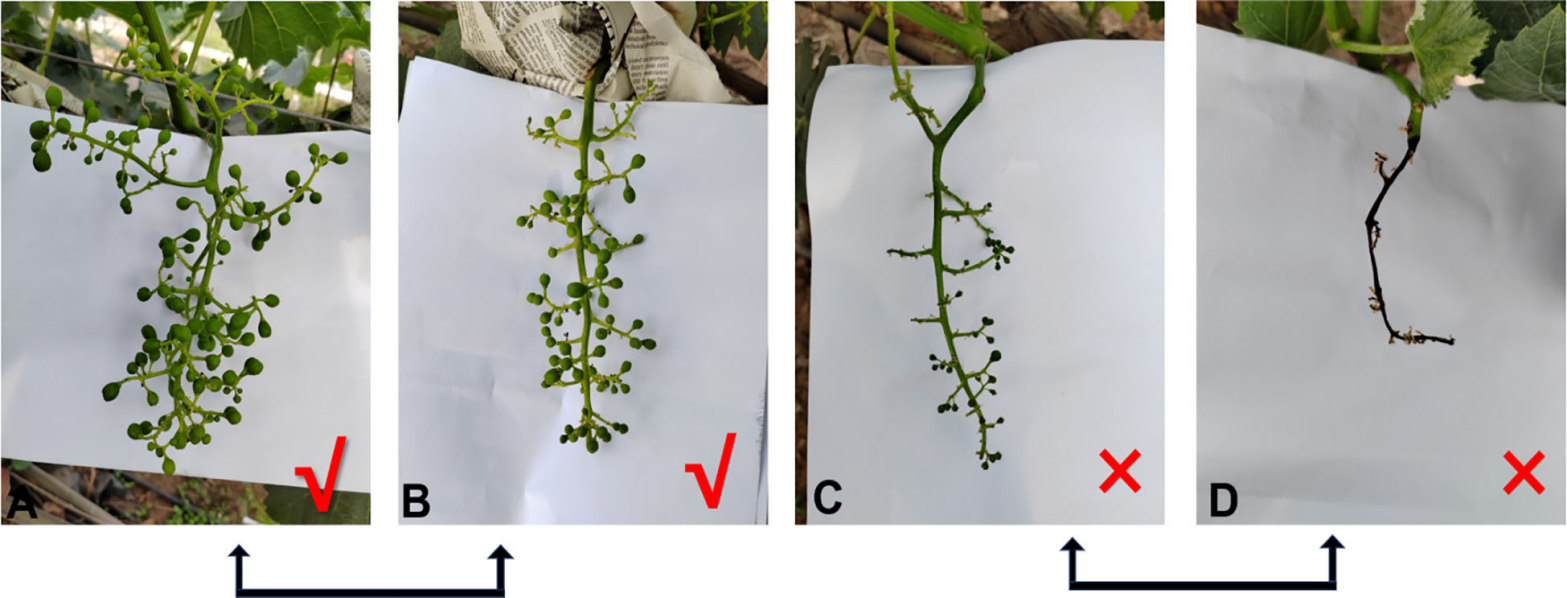
Fruit setting status of grape hybrid combinations. **(A, B)** Successful fruit setting. **(C, D)** Unsuccessful fruit setting.

The cross-combination of the ‘Shine Muscat’ grape and nine male cultivars showed significant differences in the fruit setting rate (27.8% - 62.3%). Among these, ‘Moldova’ grape exhibited the best fruit setting rate (62.3%), higher than that of other combinations (**Table 2**). This advantage was closely related to pollen vitality and storage characteristics. The pollen germination rate of the ‘Moldova’ grape collected 1 day before flowering (at the beginning of the messenger flower) was the highest (33.42%). After 7 days of storage at an ultra-low temperature of -80°C, the germination rate still remained at 30.26%, which was significantly better than that of the other cultivars (**Figures 1**, **2**). The genetic background compatibility of ‘Moldova’ grape and ‘Shine Muscat’ grape was high, promoting an efficient fertilization process. The fruit grains of the ‘Shine Muscat’ × ‘Moldova’ combination were densely distributed and full (**Figures 12A**, **B**), directly reflecting its good fruit setting effect.

**Table 2:** Fruit set rate, seed traits, and germination performance of hybrid combinations with ‘Shine Muscat’ as the female parent.

Hybrid combination	No. of tassels removed	No. of male flowers removed	No. of fruit grains	Fruit setting rate%	No. of seeds	Germination number	Germination rate%
‘Shine Muscat’ × ‘Gold Finger’	19	1303	604	46.4	48	2	4.2
‘Shine Muscat’ × ‘Moldova’	17	1267	789	62.3	72	9	12.5
‘Shine Muscat’ × ‘Shenzhou Red’	28	2281	932	40.9	165	12	7.3
‘Shine Muscat’ × ‘Queen Nina’	68	5532	2889	52.2	365	48	13.2
‘Shine Muscat’ × ‘Giant Rose’	20	1713	476	27.8	62	1	1.6
‘Shine Muscat’ × ‘Hotan Red’	8	697	207	29.7	40	5	12.5
‘Shine Muscat’ × ‘Romantic Beauty’	68	5251	2203	42.0	328	36	11.0
‘Shine Muscat’ × ‘Sweet Sapphire’	34	2955	1047	35.4	89	8	9.0
‘Shine Muscat’ × ‘Zuijinxiang’	10	530	255	48.1	123	15	12.2

The fruit setting rate of other hybrid combinations ranged from 40% to 55%, including ‘Queen Nina’ grape (52.2%), ‘Zuijinxiang’ grape (48.1%), and ‘Gold Finger’ grape (46.4%). Among these, the germination rate of ‘Queen Nina’ grape pollen after storage at -80°C for 7 days increased by nearly 80% after 5 h of culture (**Figure 7B**), showing strong vitality and stability. The fruit setting rate of ‘Zuijinxiang’ grape reached 48.1% after optimization of the pollination technology (repeated pollination for three consecutive days), indicating that the technical process could partly make up for the lack of male parent vigor.

**Figure 13:**
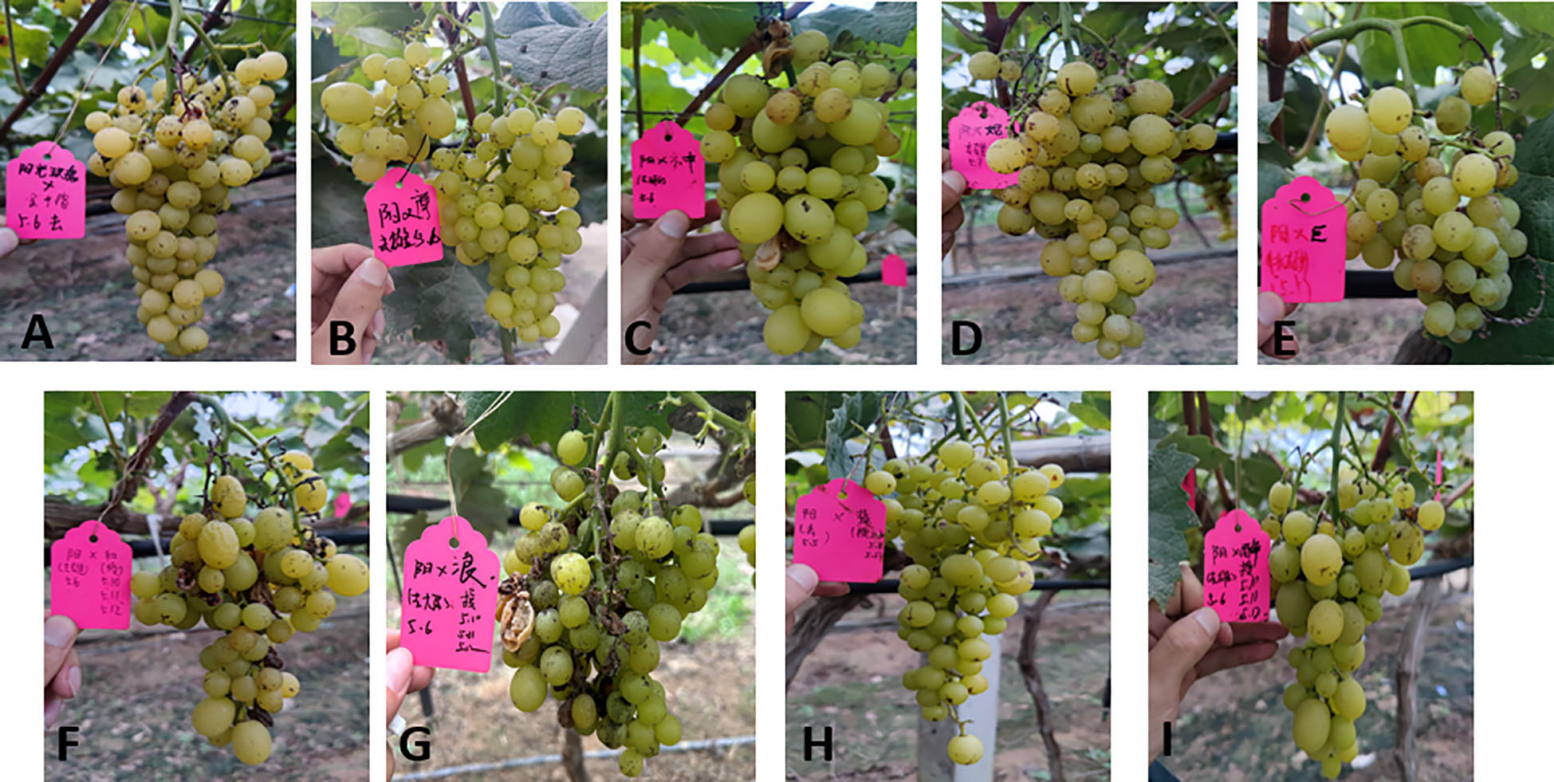
Fruit setting performance of different ‘Shine Muscat’-based grape hybrid combinations. **(A)** ‘Shine Muscat’ × ‘Gold Finger’. **(B)** ‘Shine Muscat’ × ‘Moldova’. **(C)** ‘Shine Muscat’ × ‘Shenzhou Red’. **(D)** ‘Shine Muscat’ × ‘Queen Nina’. **(E)** ‘Shine Muscat’ × ‘Giant Rose’**(F)** ‘Shine Muscat’ × ‘Hotan Red’. **(G)** ‘Shine Muscat’ × ‘Romantic Beauty’. **(H)** ‘Shine Muscat’ × ‘Sweet Sapphire’. **(I)** ‘Shine Muscat’ × ‘Zuijinxiang’.

However, the fruit setting rates of ‘Shine Muscat’ × ‘Sweet Sapphire’ (35.4%), ‘Hotan Red’ grape (29.7%), and ‘Giant Rose’ grape (27.8%) were all low. The ‘Giant Rose’ grape had the lowest fruit setting rate, which could be attributed to its low pollen vitality (germination rate of ‘Giant Rose’ grape at nonoptimal picking time was <20%; **Figure 5**). Moreover, it was sensitive to storage conditions (e.g., the vitality of ‘Giant Rose’ grape decreased significantly after 3 days of storage at 4°C), resulting in low fertilization efficiency. The fruit grains of the ‘Shine Muscat’ × ‘Giant Rose’ combination were sparse, with obvious fruit setting failures (**Figures 12C**, **D**). There were significant differences in the fruit setting rate (ranging from 27.8% to 62.3%) among the hybrid combinations of ‘Shine Muscat’ grape (as the female parent) and nine male parent cultivars, with the fruit setting status of each combination presented in **Figure 13**.

The number of seeds showed a positive correlation with the fruit setting rate, and this pattern—where parental genetic traits regulate the development and germination of hybrid seeds—aligns with findings across different crop species (Maryam et al., 2023, 2025). The germination rate of hybrid seeds was significantly influenced by parental genetic complementarity (**Table 2**). The ‘Moldova’ grape combination—with the highest fruit setting rate—had 72 seeds, whereas the ‘Giant Rose’ combination had only 62. This suggests that combinations with high fruit setting rates can provide more sufficient nutritional support for embryo development. The ‘Queen Nina’ grape combination exhibited the highest seed germination rate (13.2%), which may be associated with the genetic complementarity between the extra-large fruit traits (13–17 g; **Table 1**) of ‘Queen Nina’ and those of ‘Shine Muscat’. In contrast, the ‘Giant Rose’ combination had a seed germination rate of only 1.6%. This observation implies that pollen with low viability may cause abnormal embryo development (Wang et al., 2022b). 

**Figure 14:**
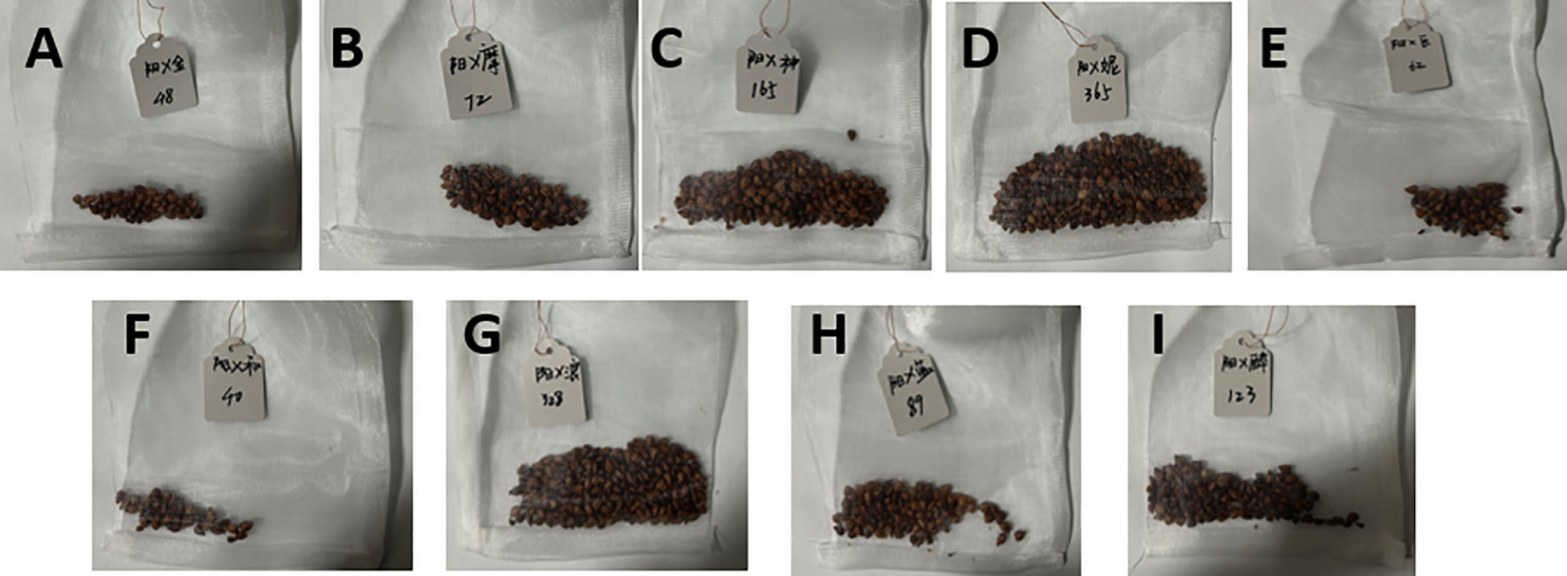
Seed harvest performance of different ‘Shine Muscat’-based grape hybrid combinations. **(A)** ‘Shine Muscat’ × ‘Gold Finger’. **(B)** ‘Shine Muscat’ × ‘Moldova’. **(C)** ‘Shine Muscat’ × ‘Shenzhou Red’. **(D)** ‘Shine Muscat’ × ‘Queen NinaQueen Nina’. **(E)** ‘Shine Muscat’ × ‘Giant Rose’**(F)** ‘Shine Muscat’ × ‘Hotan Red’. **(G)** ‘Shine Muscat’ × ‘Romantic Beauty’. **(H)** ‘Shine Muscat’ × ‘Sweet Sapphire’. **(I)** ‘Shine Muscat’ × ‘Zuijinxiang’.

**Figure 15:**
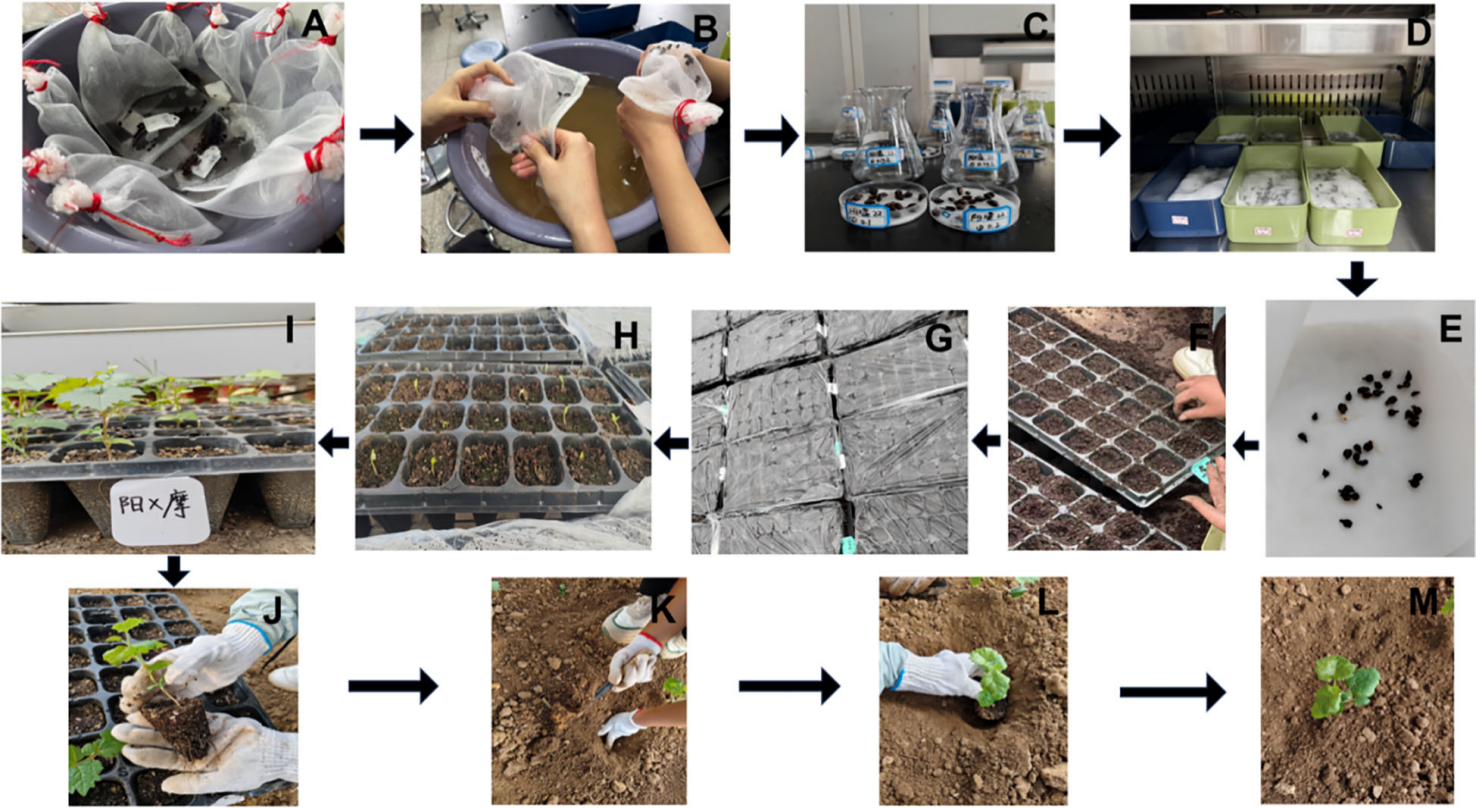
Key seedling-raising process of grape hybrid combinations with ‘Shine Muscat’ as the female parent. **(A)** Seed soaking. **(B)** Seed rubbing. **(C)** Gibberellin treatment:. **(D)** Germination promotion. **(E)** Germination. **(F)** Sowing. **(G)** Film covering. **(H)** Seedling emergence. **(I)** Seedling formation. **(J–M)** Field transplanting.

The seed harvest performance of all grape hybrid combinations using ‘Shine Muscat’ as the female parent was presented in **Figure 14**, with distinct differences in seed quantity and plumpness identified across these cross combinations. From the perspective of seed harvest, the hybrid combination of ‘Shine Muscat’ × ‘Moldova’ yielded a substantial number of plump seeds (**Figure 14B**), demonstrating robust seed development. In contrast, the ‘Giant Rose’ grape combination exhibited a notable scarcity of seeds, accompanied by significantly lower plumpness (**Figure 14E**), highlighting marked differences in seed productivity and quality between the two crosses. Further analysis of the seedling process (**Figure 15**) revealed that seeds from the high-vigor male parent combination (‘Moldova’ grape) displayed uniform emergence after soaking and germination treatments. Their seedling rate was significantly higher than that of the low-vigor combination (‘Giant Rose’ grape), which showed delayed and inconsistent germination. The results suggest that male parent traits directly influence seed plumpness, germination synchrony, and seedling establishment, underscoring the importance of parental vigor assessment in grape hybrid breeding programs (Wang et al., 2022a).

In conclusion, the success rate of hybridization was significantly improved through the standardized operation process. For ‘Zuijinxiang’ grape and other medium-vigor male parents, continuous and repeated pollination for three consecutive days could increase the fruit setting rate by 15% - 20%, compensating for the deficiency of the vitality of some male parents. The advantage of ultra-low-temperature storage was that the germination rate of pollen stored at −80°C decreased by <10% within 5 days, which was significantly better than that stored at 4°C for a short time (only for 3 days), providing technical support for cross-flowering hybridization. However, for ‘Giant Rose’ grape and other extremely low-vigor male parents, breaking through the bottleneck of compatibility by relying solely on technical optimization proved difficult, and hence the male parent cultivars should be replaced first.

### 3.7 Application effect of standardized technical process

**Figure 16:**
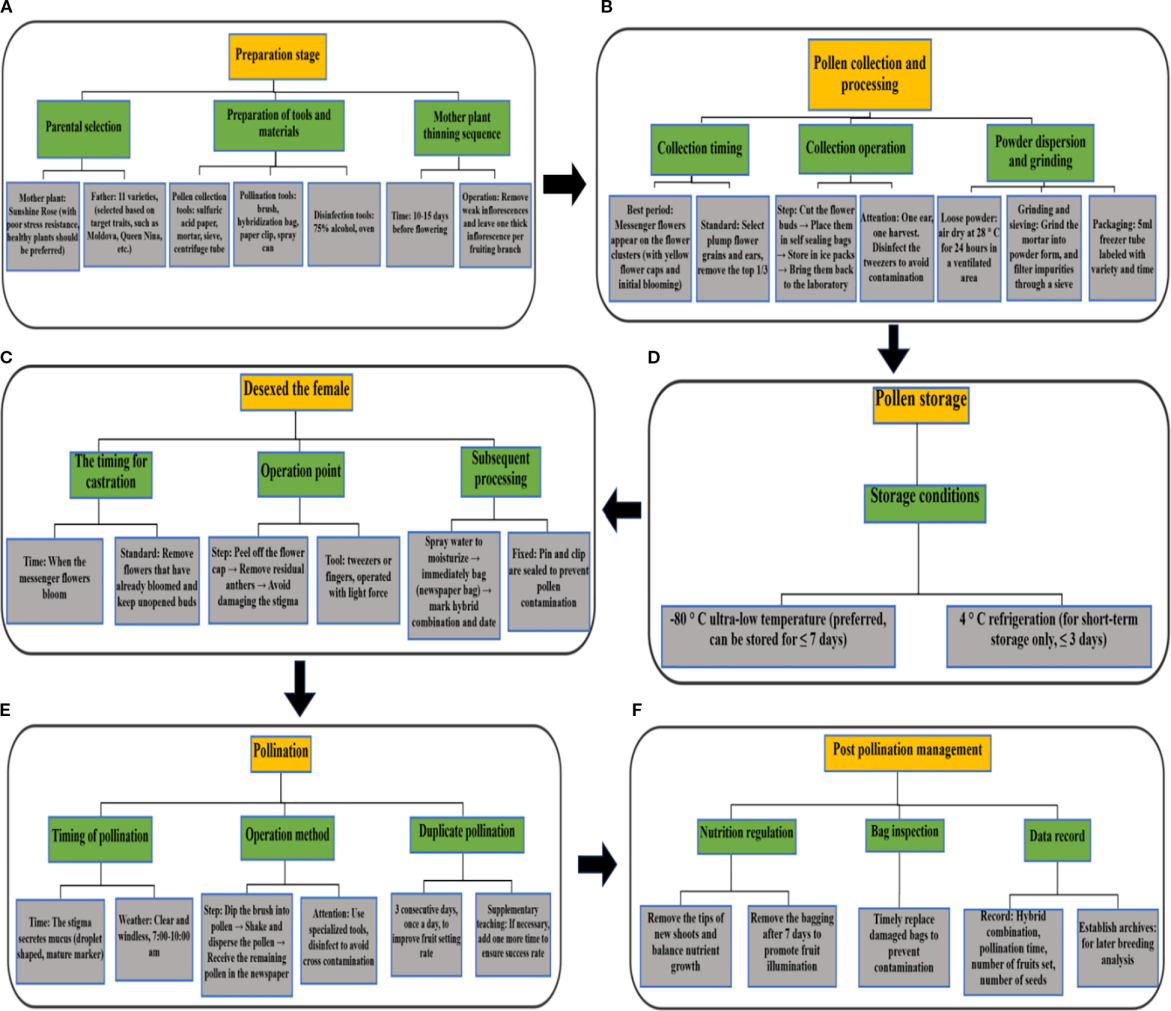
Standardized operational flowchart for grape artificial hybrid breeding **(A–F)**.

**Figure 16** displays the standardized operation technical process of grape crossbreeding, covering six core modules: parent selection, pollen processing, female parent emasculation, pollination, postpollination management, and data recording. Each module formed a reproducible, standardized process by specifying the operation time, tool requirements, and technical standards. This technical process improved the success rate of hybridization and breeding efficiency by controlling the entire process through “parent selection**-**pollen precision processing**-**emasculation pollination refinement**-**data traceability”. In practice, focusing on pollen collection at the messenger flower stage, short-term storage at -80°C, continuous repeated pollination, and parental genotype matching is crucial for improving the success rate of hybridization by more than 40%. This process effectively reduces human error by specifying the operation standards of each link, such as pollen collection time, low-temperature storage time, emasculation/pollination operation details, and so forth. It is especially suitable for large-scale breeding scenarios with a concentrated flowering period (6–10 days) and high operational intensity, providing technical support for grape hybrid breeding and new cultivar breeding in China.

## 4 Discussion

The grape flowers are small and have a is concentrated flowering period (most cultivars have a flowering period of 6–10 days), which poses challenges for large-scale hybrid pollination due to high operational intensity and technical requirements. The fruit setting rate of hybridization directly restricts breeding efficiency (Hu et al., 2015; Park et al., 2023). This study indicated that the vitality of paternal pollen, control of hybridization technology nodes, and matching of parental genotypes were the core factors affecting hybridization efficiency, consistent with previous findings (Yang, 1996).

### 4.1 Key influencing factors of pollen vitality

Pollen vitality is the foundation of successful hybridization, which is significantly influenced by the collection time and storage conditions. This study found that the germination rate of pollen collected near the flowering stage (when messenger flowers first bloomed) was the highest. For example, the germination rate of pollen collected from ‘Moldova’ grape 1 day before flowering reached 33.42%, which was significantly higher than that of immature pollen collected too early (24.58%, 7 days before flowering). This approach was slightly different from the proposal of Luo Fangmei (1985) of pollen collection 3–4 days before flowering, which might be related to the dynamic flowering period and climatic conditions of different cultivars. However, both confirmed that the stability of mature pollen cell structure and nutritional reserves were the key guarantees for vitality.

The storage temperature plays a decisive role in maintaining pollen vitality (David et al., 2020). The metabolic activity of pollen is almost stagnant under ultra-low-temperature conditions of 80°C, and after 7 days of storage, it can still maintain a high germination rate (‘Queen Nina’ grape at 24.51%). However, after 3 days of storage at 4°C, the pollen vitality of most cultivars significantly decreased (‘Gold Finger’ grape from 24.57% to 20.63%), and they almost lost germination ability after 7 days. This finding was consistent with the conclusion of Perveen and Ali (2010), who suggested that low temperatures delayed pollen aging, indicating that ultra-low-temperature storage is an effective strategy to overcome the unexpected flowering period of hybrid parents. Nevertheless, attention should be paid to shortening the storage time to reduce nutrient consumption and cell membrane damage (Ganeshan, 2015).

### 4.2 Optimization effect of hybridization technology process

The timing and precision of both male flower removal and pollination are crucial for successful hybridization. This study found that the optimal time for maternal demasculinization was when the messenger flowers bloomed. At this time, the flower buds were of moderate size, and the anthers and stigma were easily separated within the flower. Premature demasculinization can lead to immature stigma, whereas late demasculinization may result in self-pollination (He and Zhang, 1994; He et al., 1983). When removing male genitalia, peeling off the flower cap one by one and removing residual anthers are necessary. After the operation, timely water spraying and bagging isolation can minimize the risk of contamination. Pollination needs to be carried out after droplet-like secretion of mucus from the stigma (Xiong and Zhang, 2001) (a sign of maturity) and thereafter repeated for three consecutive days, increasing the fruit setting rate by 15% - 20%. This finding was consistent with the view of Hao et al. (2007). He et al. (2007) suggested that multiple pollination enhanced the chances of fertilization.

### 4.3 Decisive role of parental genotype in hybrid affinity

The difference in fruit setting rate between different paternal parents and ‘Shine Muscat’ hybrids was significant (27.8% - 62.3%), with ‘Moldova’ showing the best performance, which might be related to its strong pollen cell structure stability and outstanding stress resistance. The fruit setting rate of ‘Giant Rose’ grape was only 27.8%, which could be attributed to the sensitivity of pollen to the storage environment or low hybridization affinity. This result confirmed that the characteristic of grape hybrid affinity was regulated by genetic background (Li et al., 2009; Sabir, 2015; Puglisi et al., 2022) and, besides the conclusion of Yan et al. (2007), showing that the developmental status of maternal stigma affected fertilization efficiency, indicating that parental selection should comprehensively consider pollen vitality, stress resistance, and genetic complementarity.

## 5 Conclusions

This study focused on addressing bottlenecks in grape hybrid breeding (6-10-day concentrated flowering, strict emasculation/pollination demands, unstable pollen viability, parental affinity differences) by standardizing pollen collection-storage-pollination, offering a replicable solution. It also set forth key innovations: collect pollen at initial blooming (not 3–4 days pre-flowering), store at -80°C; fully remove anthers during emasculation and archive; screen high-affinity parents (e.g., ‘Moldova’, ‘Queen Nina’) to solve low fruit set.

## Data Availability

The raw data supporting the conclusions of this article will be made available by the authors, without undue reservation.
